# Changes in Physical Activity Associated with Mental Health in People with Type 1 Diabetes during the COVID-19 Pandemic

**DOI:** 10.3390/ijerph20043081

**Published:** 2023-02-09

**Authors:** Gabriela Correia Uliana, Daniela Lopes Gomes, Olavo Faria Galvão, Carla Cristina Paiva Paracampo

**Affiliations:** Nucleus of Behavior Theory Research, Federal University of Pará, Belém 66075-110, Brazil

**Keywords:** diabetes mellitus, social isolation, exercise, mental health, COVID-19

## Abstract

The social isolation carried out during the COVID-19 pandemic contributed to physical inactivity and impacted people’s mental health, with physical activity being an important pillar in the treatment of Type 1 Diabetes Mellitus (T1DM). Thus, this study aims to verify whether there is an association between the perception of mental health and the practice of physical activity in individuals with T1DM during social isolation in the COVID-19 pandemic in Brazil. This was a cross-sectional study conducted in July 2020, with 472 adults with T1DM, using an online form to collect sociodemographic, mental health and physical activity data during social isolation. The Chi-Square test of independence was performed with adjusted residuals analysis (*p* < 0.05). A total of 51.3% of the participants remained sedentary or stopped doing physical activity during the period of social isolation. There was an association between being interested in performing daily activities (*p* = 0.003), not feeling depressed (*p* = 0.001), feeling slightly irritated (*p* = 0.006), having slight problems with sleep (*p* = 0.012) and practicing physical activity. There was also an association between maintaining physical activity and not feeling depressed (*p* = 0.017) and feeling very slightly irritated (*p* = 0.040). Adults with T1DM who practiced physical activity during the period of social isolation due to the COVID-19 pandemic showed better aspects of mental health.

## 1. Introduction

COVID-19 is a severe acute respiratory syndrome caused by the SARS-CoV-2 virus, which had its first reported case in the city of Wuhan, China, in December 2019, and in Brazil, at the end of February 2020 [1,2]. Given the rapid spread of the disease, it is necessary to pay greater attention to people with Type 1 Diabetes Mellitus (T1DM), because even before COVID-19, studies have shown that people with T1DM are at greater risk of infections compared to people without diabetes [3,4]. Moreover, during the pandemic period, diabetes was also associated with an increased incidence and severity of COVID-19 [5].

The virus that causes COVID-19 is spread mainly through droplets expelled from the mouth or nose of infected people to people close to them. That said, social isolation was one of the measures adopted in Brazil through Law 13,979, on 6 February 2020, with the objective of separating sick or infected people, in order to avoid the contamination or spread of the virus [6]. Thus, the practice of physical activity in public places, gyms and clinics was, in general, suspended, which contributed to physical inactivity during this period, as the practice would need to be done at home. However, simple and safe exercises, such as exercises that use your own body weight, jumping rope or climbing stairs, could be done at home [7,8].

The practice of physical activity is one of the pillars of diabetes treatment, as it helps to improve glycemic control, physical conditioning, body weight control and reduce the risk of cardiovascular diseases, in addition to improving psychological well-being. The Brazilian Society of Diabetes (SBD) recommends doing more than 150 min of moderate or vigorous intensity exercise, or two to three resistance exercise sessions per week, or even combining aerobic and resistance exercises [8,9].

During the pandemic period, the importance of maintaining physical practice was also reiterated, as studies show. In the literature review carried out by Nigro et al. [10], the influence of physical activity on the activation of the immune system is reinforced, contributing to a better immune response, in addition to collaborating to reduce the negative impact of comorbidities in COVID-19 infection. Moreover, the study by De Sousa et al. [11] shows that physical activity can provide beneficial responses to pathological mechanisms.

Despite the benefits, the practice of physical activity was greatly affected during this period. In the systematic review and meta-analysis by Wunsch et al. [12], it was possible to observe that there was a reduction in physical activity during the COVID-19 pandemic in all age groups, in men and women and in most countries, with 32 of the 57 analyzed studies showing a significant decrease in physical activity. Castaneda-Babarro et al. [13] evaluated, in the Spanish population, changes in physical activity during the lockdown caused by COVID-19 and observed that there was a significant reduction in vigorous activities and walking, and an increase in sedentary time. In the systematic review and meta-analysis by Pérez-Gisbert et al. [14], studies with people with chronic diseases were analyzed, without specifying the presence of participants with T1DM, evaluating physical activity before and during the current pandemic. The results showed that in all studies there was also a reduction in physical activity levels during this period.

Social isolation had an impact not only on the practice of physical activity, but also on people’s mental health. Measures for distancing in cities increased the rates of psychological disorders, such as depression, anxiety, stress and insomnia [15,16]. In addition, people with T1DM already have a greater predisposition to have their mental health affected, with a prevalence of anxiety disorder and depression, due to the complexity of the disease and self-management to control it [17,18,19]. Thus, physical activity is even more recommended for people with T1DM, especially during the period of coping with the COVID-19 pandemic, as it can help in the prevention and treatment of psychiatric illnesses [20,21,22]. Therefore, the aim of the present study was to verify whether there is an association between the perception of mental health and the practice of physical activity by individuals with T1DM during social isolation in the COVID-19 pandemic in Brazil.

## 2. Materials and Methods

### 2.1. Type of Study

Cross-sectional, descriptive and analytical study, carried out during the period of social distancing in Brazil, in 2020, as a result of the COVID-19 pandemic. Data collection was carried out in July, using an online form, built on the Google^®^ Forms platform, in the opinion survey format, according to Resolution N.510/2016 [23]. The research was disseminated directly to people with T1DM through social networks (Whatsapp^®^, Instagram^®^ and Facebook^®^) of a research and extension project linked to a Public University in Northern Brazil.

### 2.2. Participants

The study included 472 people diagnosed with T1DM, of both genders, aged between 18 and 59 years, through convenience sampling, who agreed to participate in the research voluntarily and anonymously by checking the alternative “I read and agree to participate research” present in the first question of the questionnaire, presented after reading the Informed Consent Form (ICF). Data from 104 people who identified themselves as responsible for a minor with diabetes were excluded, such as a child/adolescent with T1DM, with diabetes T2DM, gestational [GDM], LADA, MODY, etc., or who did not complete the research questionnaire or did not agree with the ICF.

### 2.3. Instrument

An online questionnaire was used, consisting of questions developed by the study researchers, and belonging to the DSM-5 Level 1 Cross-sectional Symptom Scale [24]. The questionnaire contained 14 multiple-choice questions and a simple subjective question, referring to the age of the participants. The first two questions referred to accepting to participate in the research and being an adult with DM1; the other questions in the questionnaire were divided into three axes, namely:

(a) Sociodemographic: questions related to age, gender and region of Brazil in which they resided;
(b) Mental health: the DSM-5 Level 1 Cross-sectional Symptom Scale [24] was used, adapted to assess only questions from the psychiatric domains related to anxiety, anger, depression, sleep disorders and substance use (considering only the medication use). In the question regarding the use of medications, the use of any of the following medications on their own was considered; that is, without a medical prescription, in larger amounts or for a longer period than prescribed (e.g., analgesics (such as paracetamol, codeine), high-stimulants (such as methylphenidate or amphetamines), sedatives or tranquilizers (such as sleeping pills or diazepam) or drugs such as marijuana, cocaine or crack, synthetic drugs (such as ecstasy), hallucinogens (such as LSD), heroin, inhalants or solvents (such as cola) or methamphetamine (or other stimulants)). The scale score ranged from zero to four, with zero corresponding to mild and four corresponding to severe;
(c) Physical activity: questions regarding the practice of physical activity assessed the occurrence of physical activity before and during social isolation.

### 2.4. Ethical Issues

The research was approved by the Research Ethics Committee of the Tropical Medicine Center of the Federal University of Pará (opinion no. 4.147.663 and CAAE 32274920.0.0000.5172), meeting the legal requirements of Resolution N.466/12 [25], according to the Declaration of Helsinki.

### 2.5. Data Analysis

The Statistical Package for Social Science software, version 21, was used. Descriptive results were expressed as absolute frequency and proportion. In the analytical stage, a variable “Change in the practice of physical activity” was created, where the data “Practice of physical activity before isolation” and “Practice of physical activity during isolation” were joined in a single variable for statistical purposes and information about the difference in adherence before and during social isolation. Thus, the individuals who answered whether they practiced physical activity before (yes or no) and if they practiced physical activity during (yes or no) could be classified into: “Maintained physical activity” (yes and yes), “Maintained the physical inactivity” (no and no), “She started doing physical activity in isolation” (no and yes), “She stopped doing physical activity in isolation” (yes and no). The Chi-square test of independence was applied with adjusted residual analysis, considering the level of statistical significance of *p* < 0.05.

## 3. Results

Out of the 472 adults with T1DM evaluated, 406 (86.0%) were female, 269 (56.99%) were aged between 25 and 44 years and 222 (47%) lived in the southeast region of Brazil. During the period of social isolation, 230 (48.7%) engaged in physical activity and 242 (51.3%) remained sedentary or stopped engaging in physical activity. Table 1 shows associations between the practice of physical activity and the participants’ perception of mental health during the pandemic. It is observed in the table that having the interest or pleasure in doing things (*p* = 0.003), not feeling down, depressed, or hopeless (*p* = 0.001), feeling very slightly more irritated, grouchy, angry than usual (*p* = 0.006) and having slight problems with sleep that affected your sleep quality overall (*p* = 0.012) were associated with practicing physical activity, while having a serious lack of interest or pleasure in doing things (*p* = 0.003), feeling severely down, depressed, or hopeless (*p* = 0.001), more irritated, grouchy, angry than usual (*p* = 0.006), panicked or being frightened (*p* = 0.033), having severe problems with sleep that affected your sleep quality overall (*p* = 0.012) and having used medication moderately (*p* = 0.028) were associated with not practicing physical activity.

Table 2 shows associations between changes in physical activity practice and participants’ mental health during the pandemic. Feeling a moderate loss of interest or pleasure in doing things was associated with stopping physical activity, feeling a serious loss of interest or pleasure in doing things was inversely associated with maintaining the practice of physical activity, and not noticing changes in the interest or pleasure in doing things was inversely associated with maintaining physical inactivity during this period (*p* = 0.015).

It is possible to observe that not feeling down, depressed, or hopeless was associated with maintaining the practice of physical activity. On the other hand, feeling severely down, depressed, or hopeless was associated with maintaining physical inactivity and inversely associated with maintaining the practice of physical activity (*p* = 0.017). It was also observed that feeling very slightly more irritated, grouchy, angry than usual was associated with maintaining physical practice, while feeling severely more irritated, grouchy, angry than usual was associated with maintaining physical inactivity (*p* = 0.040) (Table 2).

## 4. Discussion

More than half of the participants (51.3%) reported remaining sedentary or that they stopped practicing physical activity during the period of social isolation. Other studies carried out in the Netherlands and Poland, respectively, with individuals with Diabetes Mellitus obtained results consistent with those of the present finding [26,27]. Ruissen et al. [27] observed that the blocking measures due to the COVID-19 pandemic resulted in lower adherence to the practice of physical activity among the public, and Grabia et al. [26] observed an increase in the percentage of people with diabetes who reported no physical activity during the pandemic (34%) compared to the pre-pandemic period (21%). Research conducted in Brazil, which evaluated the adult population in general, obtained more expressive results regarding the practice of physical activity, noting that 60.6% of the participants reported having started to practice some type of physical activity during the period of social distancing [28]. Together, these results suggest that the period of social isolation altered the practice of physical activity by people with Diabetes Mellitus.

It is noteworthy that a small percentage of participants started doing physical activity during the period of social isolation (9.5%). Knowing that having diabetes was associated with an increase in the incidence and severity of COVID-19 and that people with DM1 are at greater risk of contracting infections, it is important to emphasize the importance of physical activity in this public, as it favors better responses of the immune system, in addition to providing a smaller impact in the presence of COVID-19 infection [3,4,5,10,11].

The present study found an association between the practice of physical activity during isolation and reports of greater interest or pleasure in doing things and not feeling down, depressed, or hopeless or severely more irritated, grouchy, angry than usual, suggesting that adherence to physical activity can contribute to better mental health, and the perception of better mental health can interfere with the practice of physical activity. As this is a cross-sectional study, it is not possible to establish a cause-and-effect relationship, but the associations found are relevant for future experimental studies, as well as indicating important relationships that need to be considered by health professionals in clinical practice.

In the same direction as the results found in this study, Pieh, Budimir and Probst [22] obtained data suggesting that the practice of physical activity during social isolation due to the COVID-19 pandemic helped to improve people’s mental health, considering their quality of life, well-being, perceived stress, depressive symptoms and anxiety. Additionally, Cyranka et al. [29] observed that, for people with T1DM, isolation resulting from the pandemic proved to be a source of stress.

As a whole, the data support the hypothesis that the practice of physical activity by people with T1DM, during social isolation, contributed to the improvement of mental health, in addition to being beneficial for the treatment of the disease, as shown in the literature [8,9].

Reports of having severe problems with sleep and having moderately used medication were found to be associated with reports of not engaging in physical activity during the pandemic. Other results found in the literature show that adults with T1DM and T2DM report having a worse subjective sleep quality, shorter sleep duration, more frequent sleep disorders and more use of sleep medications, compared to adults without diabetes [30]. Reutrakul et al. [31] showed, in their systematic review with meta-analysis, that adolescents and children with T1DM have shorter sleep duration and adults with T1DM worse sleep quality, compared to people without diabetes. Considering the results of the present study and those found by Vézina-Im et al. [30] and Reutrakul et al. [31], it is suggested that physical activity can be an important variable for improving sleep in these patients, and it is recommended that it be strongly reinforced by health professionals, as the literature points out the benefits that the practice of physical activity exerts in several sleep parameters, including increased sleep duration, sleep onset latency, efficiency and quality [32,33].

This study has some limitations, such as conducting the research through an online platform, divulged directly to people with T1DM through social networks, which may have caused a bias in the study population, with the exclusion of individuals without access to social networks. Furthermore, it was not possible to evaluate the participants’ glycemic control, making it impossible to interpret more faithfully the impacts of social distancing in the practice of physical activity and mental health, in relation to glycemic control. As no other studies were found that assessed the perception of mental health and the practice of physical activity of individuals with T1DM during social isolation in the COVID-19 pandemic, this study can be considered unprecedented. Based on these observations, it is suggested that future studies seek a more homogeneous distribution of the sample and assess the glycemic control of the participants, in order to test possible associations between the practice of physical activity and mental health and their impacts on the treatment of disease, in addition to new studies that replicate the present study with patients with T1DM and individuals without diabetes, in a context of non-social isolation, so that it is possible to compare the results in relation to the investigated variables.

## 5. Conclusions

Adults with T1DM who reported better aspects of mental health, such as being interested or pleasured in doing things, not feeling down, depressed, or hopeless, feeling a little more irritated, grouchy, angry than usual and having slight problems with sleep, also reported that they practiced physical activity during the period of social isolation due to the COVID-19 pandemic. Thus, it is suggested that health professionals encourage the practice of physical activity in this public, aiming not only at improving mental health during the pandemic, but also in the post-pandemic period.

## Figures and Tables

**Table 1:** Association between physical activity and mental health in individuals with type 1 Diabetes Mellitus during social isolation in Brazil, 2020.

	Physical Activity during the Pandemic *n* (%)		*p*-Value *
	Yes	No	
Little interest or pleasure in doing things			
Nothing	36 (7.6) (+)	19 (4.0) (−)	0.003 †
Very slight	54 (11.4)	58 (12.3)	
Slight	63 (13.3)	48 (7.4)	
Moderate	51 (10.8)	71 (15.0)	
Severe	26 (5.5) (−)	46 (9.7) (+)	
Feeling down, depressed, or hopeless			
Nothing	43 (9.1) (+)	22 (4.7) (−)	0.001 †
Very slight	58 (12.3)	58 (12.3)	
Slight	59 (12.5)	48 (10.2)	
Moderate	50 (10.6)	71 (15.0)	
Severe	20 (4.2) (−)	43 (9.1) (+)	
Feeling more irritated, grouchy, angry than usual			
Nothing	28 (5.9)	21 (4.4)	0.006 †
Very slight	67 (14.2) (+)	45 (9.5) (−)	
Slight	51 (10.8)	54 (11.4)	
Moderate	58 (12.3)	71 (15.0)	
Severe	26 (5.5) (−)	51 (10.8) (+)	
Feeling nervous, anxious, frightened, worried or tense			
Nothing	25 (5.3)	18 (3.8)	0.191
Very slight	52 (11.0)	45 (9.5)	
Slight	56 (11.9)	52 (11.0)	
Moderate	60 (12.7)	72 (15.3)	
Severe	37 (7.8)	55 (11.7)	
Feeling panicked or being frightened			
Nothing	96 (20.3)	82 (17.4)	0.033 †
Very slight	48 (10.2)	57 (12.1)	
Slight	45 (9.5)	35 (7.4)	
Moderate	32 (6.8)	47 (10.0)	
Severe	9 (1.9) (−)	21 (4.4) (+)	
Avoiding situations that make you anxious			
Nothing	49 (10.4)	45 (9.5)	0.360
Very slight	53 (11.2)	65 (13.8)	
Slight	57 (12.1)	49 (10.4)	
Moderate	50 (10.6)	50 (10.6)	
Severe	21 (4.4)	33 (7.0)	
Problems with sleep that affected your sleep quality overall			
Nothing	55 (11.7)	41 (8.7)	0.012 †
Very slight	44 (9.3)	45 (9.5)	
Slight	53 (11.2) (+)	38 (8.1) (−)	
Moderate	43 (9.1)	59 (12.5)	
Severe	35 (7.4) (−)	59 (12.5) (+)	
Medication			
Nothing	150 (31.8)	142 (30.1)	0.028 †
Very slight	41 (8.7)	38 (8.1)	
Slight	22 (4.7)	20 (4.2)	
Moderate	9 (1.9) (−)	25 (5.3) (+)	
Severe	8 (1.7)	17 (3.6)	

* Chi-Squared. † Statistical Significance; Residue Analysis: (+) Significant Association (−) Negative Significant Association.

**Table 2:** Association between changes in the practice of physical activity and mental health in individuals with type 1 Diabetes Mellitus during social isolation in Brazil, 2020.

	Change in PA Practice				*p*-Value *
	Maintained the Practice of PA	Maintained Physical Inactivity	Started Doing PA in Isolation	Stopped Doing PA in Isolation	
	*N* (%)	*N* (%)	*N* (%)	*N* (%)	
Little interest or pleasure in doing things					
Nothing	28 (5.9)	6 (1.3) (−)	8 (1.7)	13 (2.8)	0.015 †
Very slight	45 (9.5)	33 (7.0)	9 (1.9)	25 (5.3)	
Slight	51 (10.8)	21 (4.4)	12 (2.5)	27 (5.7)	
Moderate	41 (8.7)	28 (5.9)	9 (1.9)	44 (9.3) (+)	
Severe	19 (4.0) (−)	25 (5.3)	7 (1.5)	21 (4.4)	
Feeling down, depressed, or hopeless					
Nothing	36 (7.6) (+)	11 (2.3)	7 (1.5)	11 (2.3) (−)	0.017 †
Very slight	45 (9.5)	27 (5.7)	13 (2.8)	31 (6.6)	
Slight	49 (10.4)	19 (4.0)	10 (2.1)	29 (6.1)	
Moderate	39 (8.3)	32 (6.8)	10 (2.1)	40 (8.5)	
Severe	15 (3.2) (−)	24 (5.1) (+)	5 (1.1)	19 (4.0)	
Feeling more irritated, grouchy, angry than usual					
Nothing	21 (4.4)	12 (2.5)	7 (1.5)	9 (1.9)	0.040 †
Very slight	57 (12.1) (+)	19 (4.0) (−)	10 (2.1)	26 (5.5)	
Slight	40 (8.5)	20 (4.2)	11 (2.3)	34 (7.2)	
Moderate	46 (9.7)	34 (7.2)	11 (2.3)	38 (8.1)	
Severe	20 (4.2) (−)	28 (5.9) (+)	6 (1.3)	23 (4.9)	
Feeling nervous, anxious, frightened, worried or tense					
Nothing	18 (3.8)	8 (1.7)	7 (1.5)	10 (2.1)	0.361
Very slight	42 (8.9)	22 (4.7)	10 (2.1)	23 (4.9)	
Slight	47 (10.0)	23 (4.9)	8 (1.7)	30 (6.4)	
Moderate	52 (11.0)	34 (7.2)	8 (1.7)	38 (8.1)	
Severe	25 (5.3)	26 (5.5)	12 (2.5)	29 (6.1)	
Feeling panicked or being frightened					
Nothing	76 (16.1)	41 (8.7)	20 (4.2)	41 (8.7)	0.161
Very slight	41 (8.7)	23 (4.9)	7 (1.5)	34 (7.2)	
Slight	37 (7.8)	15 (3.2)	7 (1.5)	21 (4.4)	
Moderate	24 (5.1)	21 (4.4)	8 (1.7)	26 (5.5)	
Severe	6 (1.3)	13 (2.8)	3 (0.6)	8 (1.7)	
Avoiding situations that make you anxious					
Nothing	41 (8.7)	21 (4.4)	8 (1.07)	24 (5.1)	0.072
Very slight	40 (8.5)	22 (4.7)	13 (2.8)	43 (9.1)	
Slight	40 (8.5)	24 (5.1)	16 (3.4)	26 (5.5)	
Moderate	45 (9.5)	26 (5.5)	5 (1.1)	24 (5.1)	
Severe	18 (3.8)	20 (4.2)	3 (0.6)	13 (2.8)	
Problems with sleep that affected your sleep quality overall					
Nothing	42 (8.9)	18 (3.8)	13 (2.8)	23 (4.9)	0.141
Very slight	39 (8.3)	19 (4.0)	5 (1.1)	26 (5.5)	
Slight	41 (8.7)	16 (3.4)	11 (2.3)	23 (4.9)	
Moderate	33 (7.0)	31 (6.6)	10 (2.1)	28 (5.9)	
Severe	29 (6.1)	29 (6.1)	6 (1.3)	30 (6.4)	
Medication					
Nothing	121 (25.6)	66 (14.0)	29 (6.1)	76 (16.1)	0.116
Very slight	34 (18.5)	14 (12.4)	6 (13.3)	25 (19.2)	
Slight	17 (3.6)	12 (2.5)	5 (1.1)	8 (1.7)	
Moderate	5 (1.1)	11 (2.3)	4 (0.8)	14 (3.0)	
Severe	7 (1.5)	10 (2.1)	1 (0.2)	7 (1.5)	

* Chi-Squared. † Statistical Significance; Residue Analysis: (+) Significant Association (−) Negative Significant Association; PA = Physical Activity.

## Data Availability

The data presented in this study are available on request from the corresponding author. The data are not publicly available due to the fact that the research was conducted through an online form that allows access to other data not used in this article.
